# Supplementary material for: Predicting visual function by interpreting a neuronal wiring diagram
Source: Nature. 2024 Oct 2;634(8032):113–23. doi: 10.1038/s41586-024-07953-5 (PMC11446822; doi:10.1038/s41586-024-07953-5)
Supplement: Supplementary file 6 — CRF and ERF predictions for individual TmY4 and TmY9 cells. Analogous to Supplementary Data 3, but for TmY target types. Shown are the top four monosynaptic pathways, the strongest pathway passing through each of the top ten intermediary types (ranking from Extended Data Fig. 7), and the trisynaptic pathway Tm1–TmY–Dm3–TmY (see the section entitled Prediction of spatial normalization). [file 41586_2024_7953_MOESM6_ESM.zip › DataS4/TmY9qΓèÑ/720575940624832747.pdf]

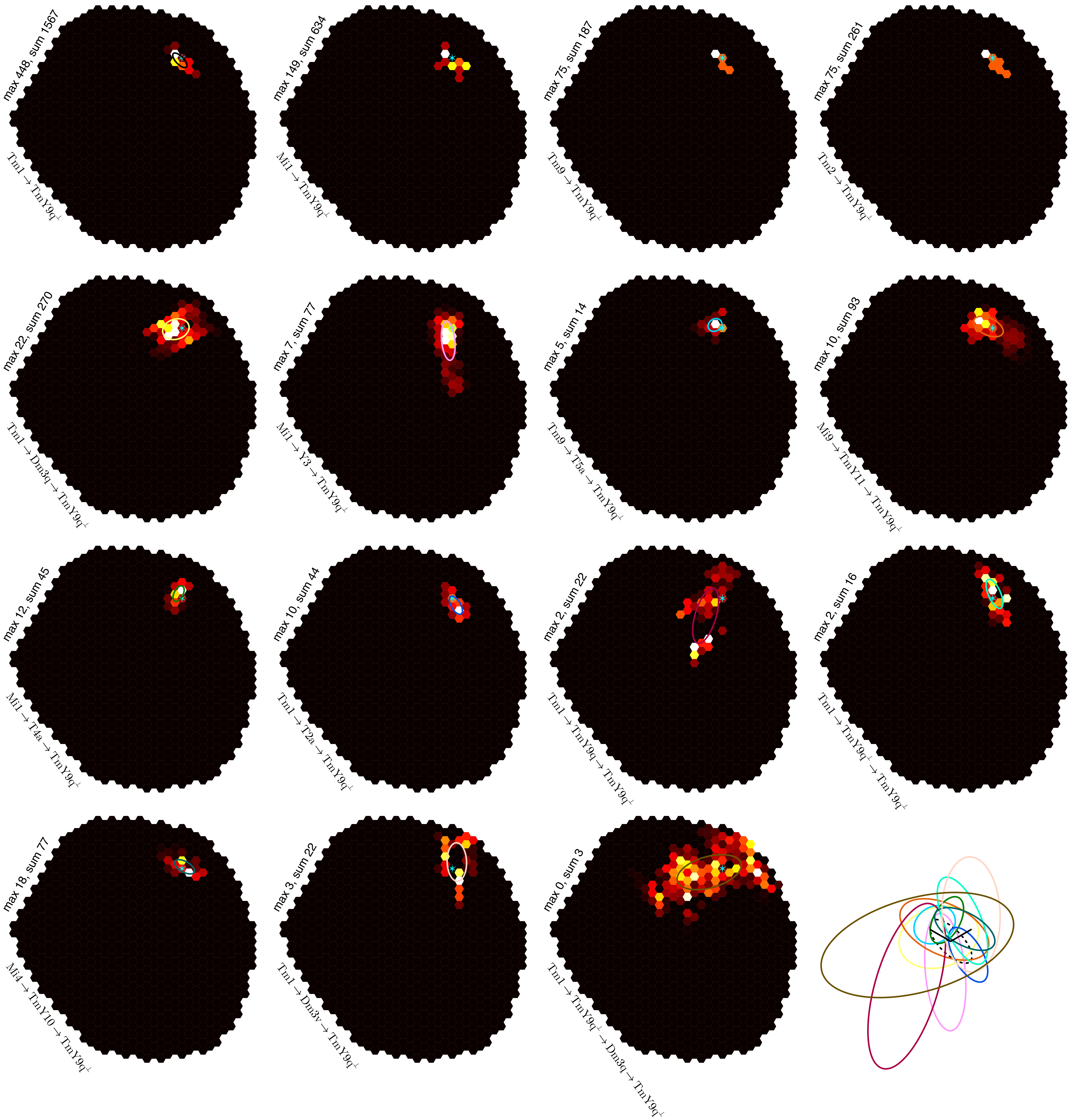
max 448, sum 1567
Tm1 → TmY9q⊥
max 149, sum 634
Mi1 → TmY9q⊥
max 75, sum 187
Tm9 → TmY9q⊥
max 75, sum 261
Tm2 → TmY9q⊥
max 22, sum 270
Tm1 → Dm3q → TmY9q⊥
max 7, sum 77
Mi1 → Y3 → TmY9q⊥
max 5, sum 14
Tm9 → T5a → TmY9q⊥
max 10, sum 93
Mi9 → TmY11 → TmY9q⊥
max 12, sum 45
Mi1 → T4a → TmY9q⊥
max 10, sum 44
Tm1 → T2a → TmY9q⊥
max 2, sum 22
Tm1 → TmY9q → TmY9q⊥
max 2, sum 16
Tm1 → TmY9q⊥ → TmY9q⊥
max 18, sum 77
Mi4 → TmY10 → TmY9q⊥
max 3, sum 22
Tm1 → Dm3v → TmY9q⊥
max 0, sum 3
Tm1 → TmY9q⊥ → Dm3q → TmY9q⊥
